# Endoscopic surveillance of extensive esophageal papillomatosis not amenable to endoscopic therapy

**DOI:** 10.1590/S1679-45082017RC3905

**Published:** 2017

**Authors:** Angelo Paulo Ferrari, Fernanda Prata Martins

**Affiliations:** 1 Hospital Israelita Albert Einstein, São Paulo, SP, Brazil.

**Keywords:** Endoscopy, Papilloma, Esophageal neoplasms, Esophagus/pathology, Case reports, Endoscopia, Papiloma, Neoplasias esofágicas, Esôfago/patologia, Relatos de casos

## Abstract

We present two cases of esophageal papillomatosis, a very rare reported disease leading to dysphagia and did not improve after endoscopic treatment. Both patients refused surgery and they were followed-up for 3 years, but no significant clinical or endoscopic changes were seen.

## RESUMO

Apresentamos dois casos de papilomatose esofágica, lesão raramente relatada, que pode levar à disfagia, e que não teve melhora após tratamento endoscópico. Ambos os pacientes recusaram cirurgia e foram acompanhados por até 3 anos, sem alterações clínicas ou endoscópicas importantes.

## INTRODUCTION

Esophageal papilloma is a benign esophageal tumor, with an estimated prevalence of up to 0.45%,^(1,2)^ that may be found in asymptomatic patients. These tumors have singular presentation of wart-like exophytic appearance. Esophageal papillomatosis, on the other hand, has been rarely found, and few cases have been reported so far.^(1,3,4)^ We present two cases in which both patients refused surgery, and they have been followed with periodical endoscopies.

## CASE REPORT

### Case 1

**Figure 1:**
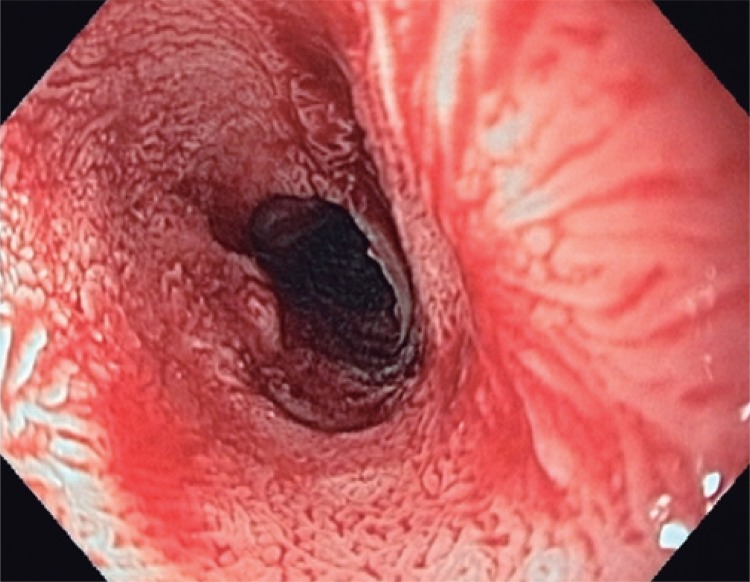
Extensive papillary lesion involving the whole esophageal circumference in patient 1

**Figure 2:**
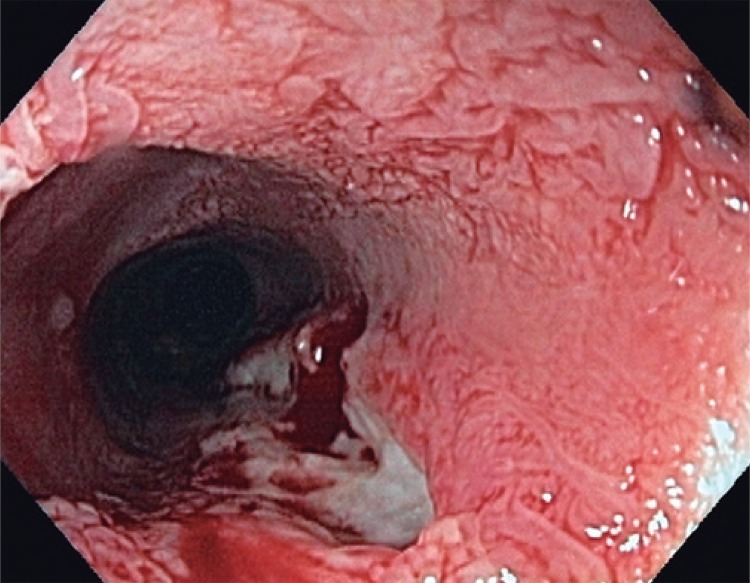
Mucosal laceration after passage of a regular gastroscope in patient 1

**Figure 3:**
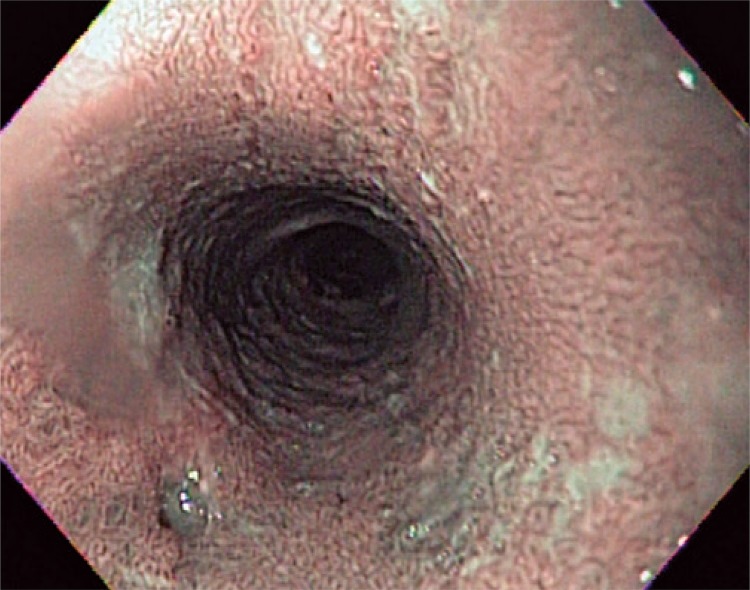
Narrow-band imaging in patient 1 showing a normal and regular vascular pattern

A 74-year-old woman complained about recurrent dysphagia for solid foods, which relieved with liquids consumption, for the last 6 months. Her past medical history was positive for mild arterial blood hypertension, without any other relevant comorbidity. She admitted to be a social drinker and smoker. The patient had lost weight, about 3kg, within the first 4 months after symptoms onset, but since then her weight has been stable. A first upper endoscopy (in February 2014) revealed an extensive papillary lesion (approximately 7 to 8cm) occupying the medium third of the esophagus (Figure 1). Introduction of a diagnostic endoscope was difficult, leading to some mucosal laceration (Figure 2). Lesion aspect resembled a colonic lateral spreading tumor. Narrow-band imaging did not show significant vascular changes (Figure 3). Biopsies showed papillary hyperplasia with paraqueratosis and hiperqueratosis associated with neutrophilic exocytosis, without signs of dysplasia, neoplasia or invasion. Therapeutic endoscopic procedure was discarded because of lesion’s extension, and patient’s refusal of surgery. She had already repeated upper endoscopy in five different occasions (March and November 2014, February and November 2015). In each procedure the endoscopic aspect presented no significant changes, regarding lesion extension and difficulty in passing the gastroscopy across the lesion. Multiple biopsies repeated at each procedure did not show differences in histological findings, and none of them revealed signs of dysplasia or neoplasia.

### Case 2

**Figure 4:**
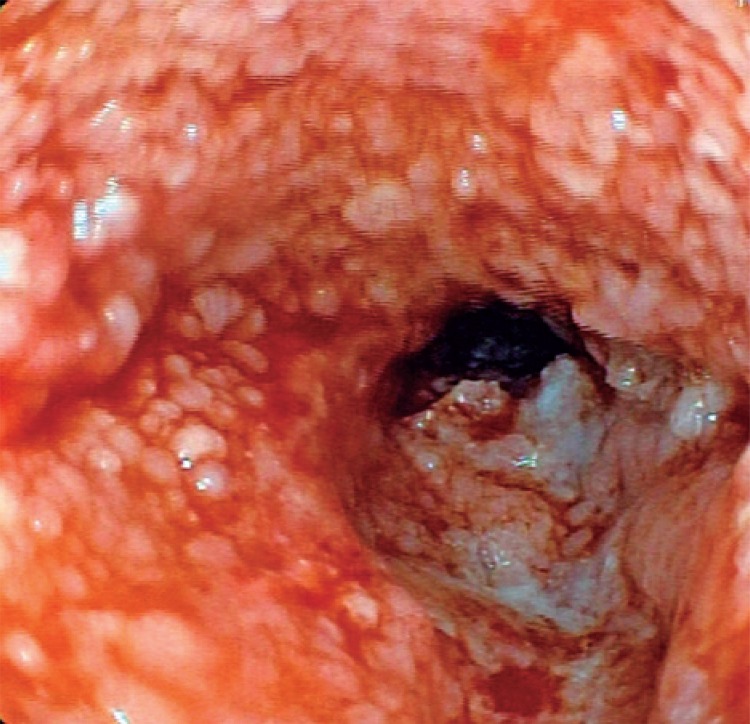
Lesion involving the whole esophageal circumference

A 73-year-old woman with history of dysphagia, and mild diabetes controlled with drugs and diet. The patient was also referred to endoscopy. She reported social drinking, and denied smoking. At the procedure a long papillary esophageal lesion, with about 10 to 11cm in length was noted in the middle portion of the esophagus, proximally involving part of the circumference, but progressing to involve the whole circumference (Figure 4). The distal esophagus was normal. Biopsies showed the same aspect of esophageal papillomatosis seen in the patient 1. The patient 2 refused surgical treatment and she has been followed with regular 6-month endoscopies for 3 years now, but no changer have been seen in endoscopic and histological aspects.

## DISCUSSION

Esophageal papilloma is believed to be associated to human papillomaviruses or to chronic mucosal irritation,^(2,5)^ however, this association is so far considered controversial. Esophageal papillomas are easily recognized lesion, and endoscopically removed without difficulty using different accessories (biopsy forceps or snares) regardless of the size of the lesion. Recurrence may also occur. Malignant potential has been suspected.^(2,6)^

Esophageal papillomatosis was first described in 1995^(7)^ associated with adenocarcinoma. Papillomatosis etiology is not defined and multiple factors have been associated with the disease, such as human papillomaviruses, gastroesophageal reflux, endoscopic esophageal therapies (sclerotherapy, dilatation and stents). Except for the papillomaviruses that was not evaluated in our two patients, other factors were absent, except smoking that is also considered a contributing factor, which is little described in the literature.

Although the main recommendation is the removal of all papilloma lesions, in our two cases, because of the extension of several centimeter involving the whole esophageal circumference, this option was considered impossible. Total esophagectomy was proposed to both patients, who were alerted to the risk of carcinoma development. Both patients chose to continue under strict endoscopic surveillance, so far about 3 years, but no changes have been seen in clinical, endoscopic and histological findings. In order to optimize neoplasia recognition, narrow-band imaging has been applied in every endoscopy to look for abnormal vascular patterns. Recently, both patients underwent a thoracic computed tomography and no signs were found of involvement outside the esophagus. Lymph nodes were absent.

A discussion was carried out about performing endoscopic ultrasound, but because of the difficult for passing the regular gastroscope, including some mucosal laceration, we avoided to pass an echo endoscope.
